# Trend of Cutaneous Leishmaniasis in Jordan From 2010 to 2016: Retrospective Study

**DOI:** 10.2196/14439

**Published:** 2020-03-24

**Authors:** Mohammad Alhawarat, Yousef Khader, Bassam Shadfan, Nasser Kaplan, Ibrahim Iblan

**Affiliations:** 1 Jordan Field Epidemiology Training Program Jordan Ministry of Health Amman Jordan; 2 Jordan University of Science and Technology Irbid Jordan; 3 Parasitic and Zoonotic Disease Department Communicable Disease Directorate Ministry of Health Amman Jordan

**Keywords:** cutaneous leishmaniasis, incidence, Jordan

## Abstract

**Background:**

Cutaneous leishmaniasis (CL) is endemic in the Middle East, with countries such as Syria reporting high incidence rates.

**Objective:**

This study aimed to assess the trends in the incidence of cutaneous leishmaniasis (CL) in Jordan from 2010 to 2016.

**Methods:**

This retrospective study included all cases of CL that had been reported to the Leishmaniasis Surveillance System in the Department of Communicable Diseases at the Jordan Ministry of Health during the period from 2010 to 2016. A total of 1243 cases were reported and met the case definition.

**Results:**

A total of 1243 cases (60.65% [754/1243] males and 39.34% [489/1243] females) were diagnosed during the study period. Of this sample, 233 patients (19.13%) were aged <5 years old, 451 (37.03%) were aged between 5-14 years old, 190 (15.60%) were aged between 15-24 years old, and 344 (28.24%) were aged ≥25 years old. Of those, 646 (51.97%) were Jordanians and 559 (44.97%) were Syrians. The average annual incidence rate of 1.70 per 100,000 people between 2010 and 2013 increased to 3.00 per 100,000 people in the years 2014 to 2016. There was no difference in incidence rates between Jordanians and Syrian refugees between 2010 and 2012. After 2012, the incidence rate increased significantly among Syrian refugees from 1.20 per 100,000 people in 2012 to 11.80 per 100,000 people in 2016. On the contrary, the incidence rate did not change significantly among Jordanians.

**Conclusions:**

The incidence rate of leishmaniasis in Jordan has increased in the last three years because of the influx of Syrian refugees into Jordan. A massive effort toward reservoir and vector control, along with actively pursuing diagnosis in endemic foci, will be helpful. Proper and studious reporting of cases is also a necessity for the eradication of this disease.

## Introduction

### Background

Leishmaniasis is a vector-borne disease that is transmitted via female sandflies and caused by an intracellular protozoon called Leishmania. It is endemic in 98 countries and 3 continents [1]. Out of 30 mammal-infecting Leishmania species, 21 are known to infect humans [1]. Leishmaniasis is subdivided into 3 types: cutaneous, mucocutaneus, and visceral. Cutaneous leishmaniasis (CL) is the most common type, and almost 95% of its cases are found in North and South America, the Mediterranean region, the Middle East, and Central Asia [2]. It usually manifests in the form of skin lesions, particularly ulcers, which leave permanent scars and cause severe disability. An estimate of around 0.7 to 1.3 million new cases are reported each year, and more than two-thirds of the new cases are found in 6 countries: Afghanistan, Algeria, Brazil, Colombia, Iran, and Syria [2].

Poverty, overcrowding, immigration, and other risk factors have a great role in increasing the incidence of CL. There are currently no available drugs or vaccines to prevent infections, and despite numerous preventative measures, leishmaniasis remains an important, neglected, zoonotic disease and a big challenge to public health, especially in underdeveloped countries [3]. The trends of CL vary from one country to another in the Middle East. The incidence rate has decreased in Saudi Arabia, whereas it has increased in Iraq and Syria, especially during the civil war [4]. In general, previous studies in the Middle East have shown that males are more likely to be affected with CL [5-7].

In Jordan, it has been an emerging disease since the 1980s, and is still an important public health problem despite existing control and prevention measures. In a previous study in the last decades, the incidence rate of CL in Jordan has been shown to be increasing [8]. One study had assessed the spatial and temporal characteristics of CL in the years from 1999 to 2010, pre–Arab Spring in Jordan and Syria, and it showed that the risk of CL varied both spatially and temporally in both countries [9]. That study showed that the patterns of the disease in Jordan could be described as relatively low and heterogeneous whereas those in Syria were relatively much higher and less heterogeneous.

### Objectives

The CL surveillance system in Jordan receives reports on a weekly basis from 21 reporting sites from all districts and governorates in the country. This study aimed to assess the trend in the incidence of CL in Jordan from 2010 to 2016.

## Methods

This retrospective study included all cases of CL that had been reported to the Leishmaniasis Surveillance System in the Department of Communicable Diseases at the Jordan Ministry of Health during the period from 2010 to 2016. A total of 1243 cases were reported and met the case definition.

A suspected case is defined as a person who was in Jordan during the study period and showed clinical signs (skin lesions) of infection, wherein the papule appears and may enlarge to become an indolent ulcerated nodule. A confirmed case is a person who was in Jordan during the study period and showing clinical signs of infection, with paracytological confirmation of the diagnosis by a positive smear or culture from a skin lesion [10].

The necessary and available data were retrieved from the surveillance system. Data included patient’s age, gender, address, nationality, occupation, reporting site, reporting month, reporting year, and location of lesion. The Ethical Committee at the Ministry of Health approved the study. Data were entered into an Excel (Microsoft, Redmond, Washington, United States) file and analyzed using SPSS Statistics for Windows, Version 23.0 (IBM Corporation, Armonk, New York, United States).

## Results

### Patients’ Characteristics

A total of 1243 CL cases were reported. Of the total reported cases, 754 (60.65%) were males, 489 (39.34%) were females, and the mean age of the patients was 18.6 years old (SD 16.8). Overall, 233 (19.13%) patients were aged <5 years old, 451 (37.03%) were aged between 5-14 years old, 190 (15.60%) were aged between 15-24 years old, and 344 (28.24%) were aged ≥25 years old. Half of reported cases were from the southern region of the country (Table 1). A total 563 (45.29%) patients had head lesions, 186 (14.96%) had trunk lesions, 382 (381.60%) had leg lesions, and 426 (34.27%) had arm lesions.

**Table 1:** Distribution of leishmaniasis cases in Jordan from 2010 to 2016 by age, gender, and region.

Characteristics		Frequency, n (%)
**Age (years)**		
	0-4	233 (19.1)
	5-14	451 (37.0)
	15-24	190 (15.6)
	≥25	344 (28.2)
**Gender**		
	Male	754 (60.7)
	Female	489 (39.3)
**Region**		
	South	575 (49.4)
	North	179 (15.4)
	Middle	410 (35.2)

### Incidence and Trend

**Figure 1:**
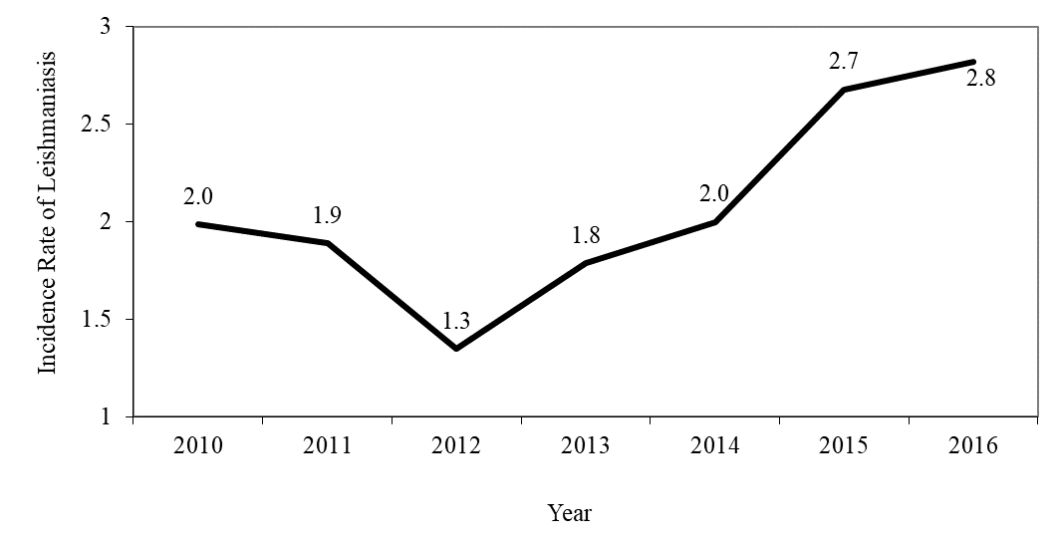
The trend of the overall incidence rate of cutaneous leishmaniasis per 100,000 people in Jordan from 2010 to 2016.

**Figure 2:**
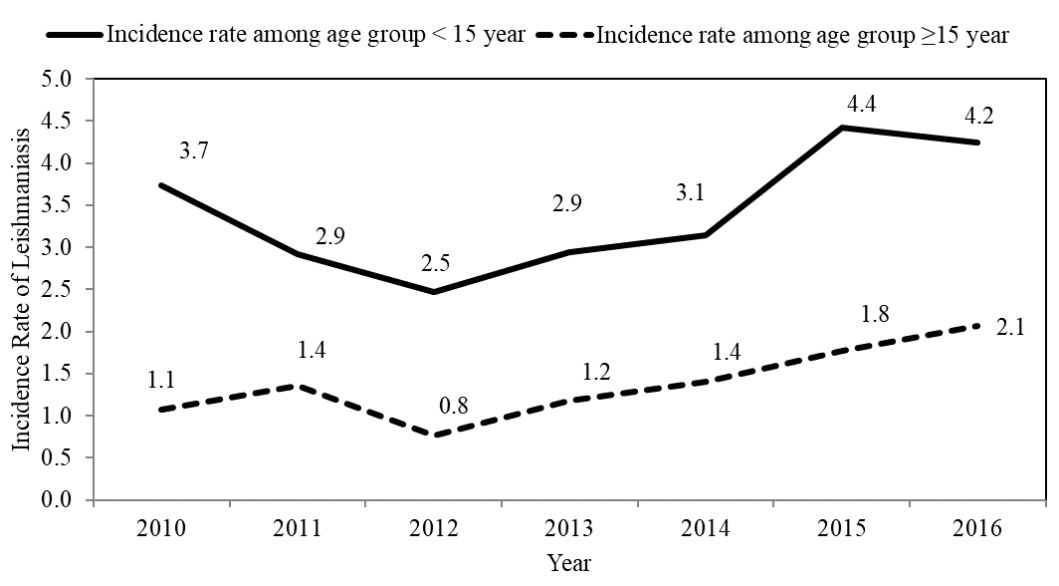
The incidence rate of cutaneous leishmaniasis per 100,000 people in Jordan by age categories from 2010 to 2016.

**Figure 3:**
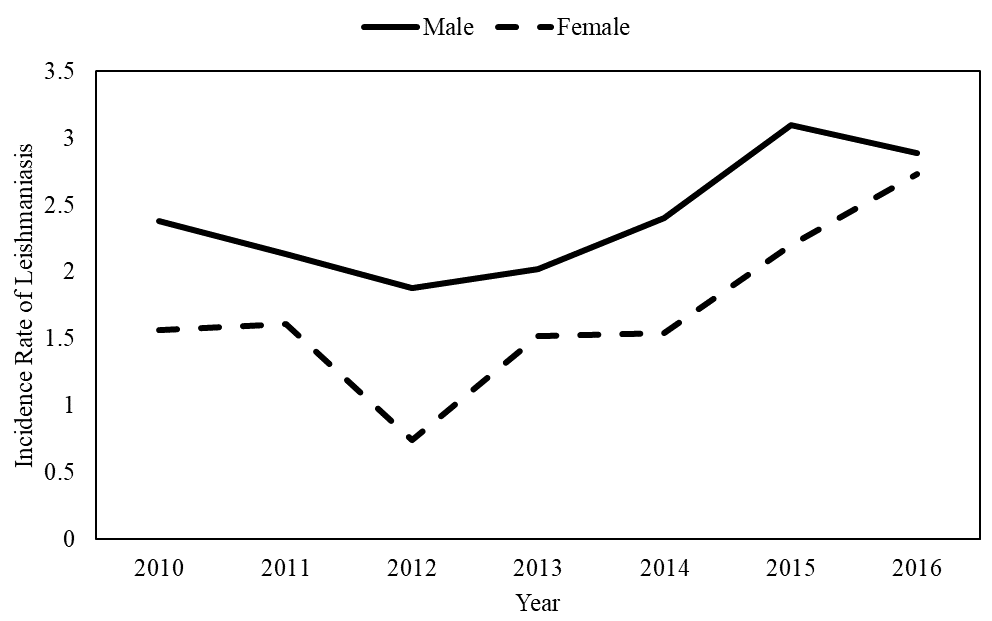
The incidence rate of cutaneous leishmaniasis per 100,000 people in Jordan by gender from 2010 to 2016.

**Figure 4:**
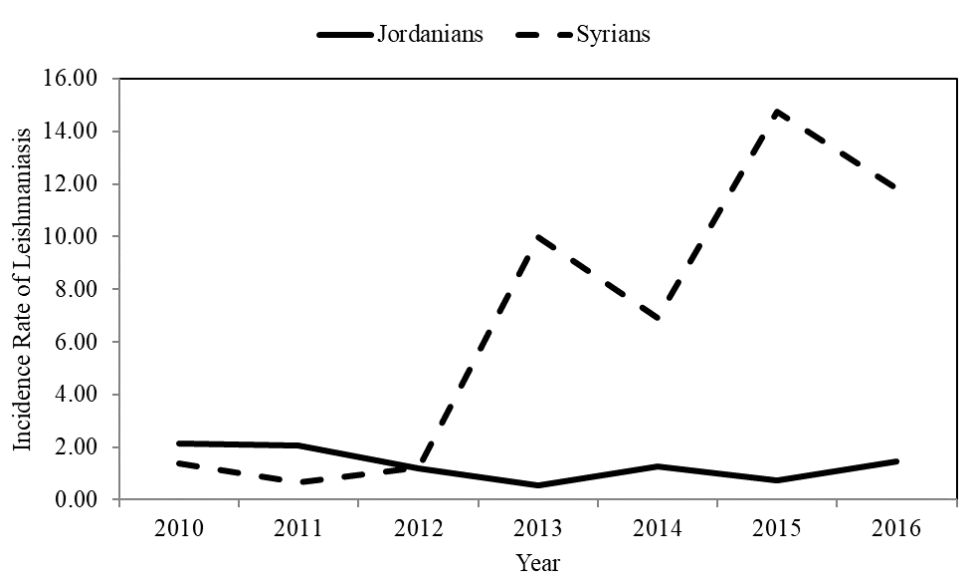
The incidence rate of cutaneous leishmaniasis per 100,000 people in Jordan by nationality from 2010 to 2016.

The average annual incidence rate was 1.70% among 100,000 people during the period from 2010 to 2013. In the period from 2014 to 2016, when Syrian refugees entered the country, the average incidence rate increased to 3.00% in every 100,000 people (Figure 1). The incidence rate was higher among those aged less than 15 years old compared with those aged ≥15 years old (Figure 2), and it was higher among males compared to females in all studied years. In both genders, the incidence decreased during the period from 2010 to 2012, following which it started to increase again (Figure 3). In total, 646 (51.97%) of reported leishmaniasis cases were Jordanians and 559 (44.97%) were Syrians. There was no difference in incidence rates between Jordanian and Syrian refugees between 2010 and 2012, but after 2012, the incidence rate increased significantly among Syrian refugees from 1.20 per 100,000 people in 2012 to 11.80 per 100,000 people in 2016. On the contrary, the incidence rate did not change significantly among Jordanians (Figure 4).

## Discussion

### Principal Findings

This study assessed changes in the incidence of CL in Jordan between 2010 and 2016. Males were predominant among affected cases in all age categories, and this finding was also reported in other countries, including Saudi Arabia and Iran [5-7]. The increased infection rates among males in Jordan might be explained by the fact that males are usually responsible for outdoor work and work in farms. Moreover, most females in Jordan traditionally cover most parts of their bodies, thus they are fairly well protected from sandflies.

During the study period, the highest incidence rate was among subjects aged less than 15 years old. This finding is consistent with the findings from a longitudinal study in the endemic area in the eastern region of Saudi Arabia [11], and with the findings of other studies in Saudi Arabia and Iran [5-7]. This finding is probably explained by the fact that children spend more time outdoors, and therefore are more likely to be exposed to sandfly bites. However, other studies in Saudi Arabia [12], Iran [13], and Kuwait [14] have shown that people aged between 21-30 years old are the most susceptible because most laborers are in this age group.

On the basis of the geographic distribution of CL, the southern region of Jordan was an endemic area. However, the Zarqa governorate is now a new hot reporting site because of the presence of the Syrian refugees’ camp in this governorate. In agreement with the findings of studies in Lebanon [15] and Turkey [16], this study showed an increasing trend in the incidence rate of CL in Jordan during the study period. The increased rate of CL in Jordan in the past few years is explained by an increasing number of Syrian refugees in Jordan over time. Poor housing, absence of clean water, inadequate sanitation, deficient medical facilities and services, and abundant sandfly populations have contributed to CL among Syrian refugees.

CL emerged in areas where displaced Syrians and disease reservoirs coexist. In 2013, 1033 new cases were reported in Lebanon, of which 96.6% occurred among the displaced Syrian refugee populations [15]. In Turkey, nonendemic parasite strains (*Leishmania major* and *Leishmania donovani*) were introduced by incoming refugees [16]. Other studies in Saudi Arabia and Iran have shown a decline in the number and incidence rate of CL in the same period [5,6]. The main limitation of this study is the underreporting of CL cases.

### Conclusions

In conclusion, CL is increasing in Jordan, especially after the Syrian war, but countries with ample resources, like Jordan, have taken measures to control the spread of the disease. However, challenges still remain to be solved because of huge refugee movement into the country. A massive effort toward reservoir and vector control, along with actively pursuing diagnosis in endemic foci, will be helpful. Proper and studious reporting of cases is also a necessity for the eradication of this disease, as health care practitioners rely on these data for framing health policies. Future research is needed to determine the main risk factors contributing to the increasing trend of the occurrence of leishmaniasis, and to implement and evaluate control and prevention measures in Jordan. Moreover, there is an urgent need for developing national health policies and action plans for combating CL in Jordan.

## Abbreviations

CL: cutaneous leishmaniasis

## References

[1] Centers for Disease Control and Prevention.

[2] World Health Organization.

[3] Pace D (2014). Leishmaniasis. J Infect.

[4] Salam N, Al-Shaqha WM, Azzi A (2014). Leishmaniasis in the middle East: incidence and epidemiology. PLoS Negl Trop Dis.

[5] Amin TT, Al-Mohammed HI, Kaliyadan F, Mohammed BS (2013). Cutaneous leishmaniasis in Al Hassa, Saudi Arabia: epidemiological trends from 2000 to 2010. Asian Pac J Trop Med.

[6] Khosravani M, Moemenbellah-Fard MD, Sharafi M, Rafat-Panah A (2016). Epidemiologic profile of oriental sore caused by Leishmania parasites in a new endemic focus of cutaneous leishmaniasis, southern Iran. J Parasit Dis.

[7] Khazaei S, Hafshejani AM, Saatchi M, Salehiniya H, Nematollahi S (2015). Epidemiological aspects of cutaneous leishmaniasis in Iran. Arch Clin Infect Dis.

[8] Khoury S, Saliba EK, Oumeish OY, Tawfig MR (1996). Epidemiology of cutaneous leishmaniasis in Jordan: 1983-1992. Int J Dermatol.

[9] Jaber SM, Ibbini JH, Hijjawi NS, Amdar NM (2014). An exploratory comparative study of recent spatial and temporal characteristics of cutaneous leishmaniasis in the Hashemite kingdom of Jordan and Syrian Arab Republic pre-Arab spring and their health policy implications. Appl Spat Anal Policy.

[10] World Health Organization.

[11] Al-Tawfiq JA, AbuKhamsin A (2004). Cutaneous leishmaniasis: a 46-year study of the epidemiology and clinical features in Saudi Arabia (1956-2002). Int J Infect Dis.

[12] Elmekki MA, Elhassan MM, Ozbak HA, Qattan IT, Saleh SM, Alharbi AH (2017). Epidemiological trends of cutaneous leishmaniasis in Al-Madinah Al-Munawarah province, western region of Saudi Arabia. J Glob Infect Dis.

[13] Karami M, Doudi M, Setorki M (2013). Assessing epidemiology of cutaneous leishmaniasis in Isfahan, Iran. J Vector Borne Dis.

[14] Al-Taqi M, Behbehani K (1980). Cutaneous leishmaniasis in Kuwait. Ann Trop Med Parasitol.

[15] Alawieh A, Musharrafieh U, Jaber A, Berry A, Ghosn N, Bizri AR (2014). Revisiting leishmaniasis in the time of war: the Syrian conflict and the Lebanese outbreak. Int J Infect Dis.

[16] Koltas IS, Eroglu F, Alabaz D, Uzun S (2014). The emergence of Leishmania major and Leishmania donovani in southern Turkey. Trans R Soc Trop Med Hyg.

